# Validity and reliability study of the Moral Distress Questionnaire in Turkish for nurses

**DOI:** 10.1590/1518-8345.2960.3319

**Published:** 2020-08-12

**Authors:** Sebnem Cinar Yucel, Eda Ergin, Fatma Orgun, Mücahide Gokçen, Ismet Eser

**Affiliations:** 1 Ege University, Nursing School, İzmir, Turkey.; 2 İzmir Bakircay University, Faculty of Health Sciences, İzmir, Turkey.

**Keywords:** Morale, Ethics, Nursing, Reliability and Validity, Nurses, Surveys and Questionnaires, Moral, Ética, Enfermagem, Confiabilidade e Validade, Enfermeiras e Enfermeiros, Inquéritos e Questionários, Moral, Ética, Enfermería, Confiabilidad y Validez, Enfermeras y Enfermeros, Encuestas y Cuestionarios

## Abstract

**Objective:**

to determine the validity and reliability of the Turkish language version of the Moral Distress Questionnaire for nurses.

**Method:**

methodological study whose sample consisted of 200 nurses working in the internal medicine and surgery clinics of a university hospital. Data was collected using the personal information form and the Moral Distress Questionnaire for nurses.

**Results:**

in the Main Components Analysis, the items were grouped under three factors. Findings regarding confirmatory factor analysis: chi-square goodness: 2.28, goodness of fit index: 0.88, comparative fit index: 0.88, non-normed fit index: 0.86, root mean square error of approximation: 0.07. The Cronbach’s alpha coefficient was found to be 0.79 as a result of the analysis conducted in order to test the internal consistency of the scale. It was seen that these three factors explained 44.92% of the total variance.

**Conclusion:**

in this present study, the Turkish version of the Moral Distress Questionnaire was found to be valid and reliable for the Turkish society. It is recommended that the Moral Distress Questionnaire for nurses should be used in future studies to be conducted with nurses in order to investigate of issues of ethical dilemma.

## Abstract (pt)

**Objetivo:**

determinar a validade e a confiabilidade da versão em idioma turco do *Moral Distress Questionnaire* para enfermeiros.

**Método:**

estudo metodológico cuja amostra foi composta por 200 enfermeiros atuantes nas clínicas de medicina interna e cirurgia de um hospital universitário. Os dados foram coletados por meio do formulário de informações pessoais e do *Moral Distress Questionnaire* para enfermeiros.

**Resultados:**

na Análise de Componentes Principais, os itens foram agrupados em três fatores. Resultados da análise fatorial confirmatória: qualidade do ajuste do qui-quadrado: 2,28, índice de qualidade do ajuste: 0,88, índice de ajuste comparativo: 0,88, índice de ajuste não normatizado: 0,86, raiz quadrada da média do erro de aproximação: 0,07. O coeficiente alfa de Cronbach foi de 0,79 como resultado da análise realizada para testar a consistência interna da escala. Observou-se que esses três fatores explicaram 44,92% da variância total.

**Conclusão:**

neste estudo, a versão turca do *Moral Distress Questionnaire* foi considerada válida e confiável para a sociedade turca. Recomenda-se que o *Moral Distress Questionnaire* para enfermeiros seja utilizado em estudos futuros a serem conduzidos, com esses profissionais, para investigar questões de dilema ético.

## Abstract (es)

**Objetivo:**

determinar la validación y confiabilidad de la versión en idioma turco del *Moral Distress Questionnaire* para enfermeros.

**Método:**

estudio metodológico cuya muestra estuvo conformada por 200 enfermeras que trabajaban en clínicas médicas y de cirugía internas de un hospital universitario. Los datos se recopilaron utilizando el formulario de información personal y el Cuestionario de Estrés Moral para enfermeros.

**Resultados:**

en el Análisis de Componentes Principales, los ítems se agruparon en tres factores. Resultados del análisis factorial confirmatorio: calidad de ajuste de chi-cuadrado: 2,28, índice de bondad de ajuste: 0,88, índice de ajuste comparativo: 0,88, índice de ajuste no normalizado: 0,86, raíz cuadrada del error de aproximación promedio: 0.07. Se determinó que el coeficiente alfa de Cronbach fue de 0,79 como resultado del análisis realizado para comprobar la consistencia interna de la escala. Se observó que estos tres factores explicaban el 44,92% de la varianza total.

**Conclusión:**

en el presente estudio, la versión turca del Cuestionario de Estrés Moral resultó ser válida y confiable para la sociedad turca. Se recomienda que el Cuestionario de Estrés Moral para enfermeros se utilice en futuros estudios, con estos profesionales, para investigar cuestiones de dilema ético.

## Introduction

The concept of moral nuisance/distress was described for the first time in 1984 as “a distress experienced when a health care professional knows the right action to be taken; but it is almost impossible to do the right action due to institutional obstacles”^(^ 1 ^)^. The nursing literature^(^ 2 ^)^ described moral distress as one of the main ethical problems affecting nurses in all health care systems and defined it as a threat to the integrity of nurses and to the quality of patient care. Therefore, moral distress endangers the ability of nurses to achieve optimal patient care and to obtain high-quality outcomes for patients and, besides, nurses who have moral distress may experience burnout and eventually leave their work^(^ 3 ^)^.

In recent years, the reconstruction of health care systems around the world, the reduction in inpatient capacity, the increasing awareness about patient rights, the reforms to increase productivity in the healthcare system and the rapid improvement in therapeutical technologies and pharmacological initiatives have made the implementation of nursing care more and more complex, causing an increase the moral distress and ethical climate experienced by nurses^(^ 4 ^)^. In the process of ethical decision making and implementation by nurses, business friendship, ethicalness of the working environment, physician group, and the style of managers are influential. On one hand, the existence of moral principles that constitute the personal characteristics of nurses and, on the other hand, the existence of ethical principles and the situation of experienced distress form a basis to the situation named moral distress^(^ 5 ^)^.

The moral distress experienced by health care professionals in health care environments is related to many factors^(^ 1 ^)^. In the studies conducted, moral distress at an individual level has been associated with depression, anger, guilt, anxiety, shame, sadness, feelings of failure, despair and pain^(^ 6 ^-^ 9 ^)^. Lack of communication and cooperation between team members, different perspectives of professionals on ethical issues, limited resources, increased workload due to personnel insufficiency, inconsistency between institutions and health policies, lack of administrative support and a negative ethical climate are among the most important reasons of moral distress at an institutional level^(^ 4 ^-^ 10 ^)^.

Several measurement tools for investigating the effect of ethical dilemmas on stress have been reported in the literature^(^ 7 ^,^ 9 ^,^ 11 ^-^ 13 ^)^. These tools include the Moral Distress Scale which assesses moral distress among nurses^(^ 7 ^)^; the Moral Distress Assessment Questionnaire which assesses moral distress experiences in terms of frequency, type, intensity, and duration^(^ 11 ^)^; the Stress of Conscience Questionnaire which assesses a measurement of stress arising from a disturbed conscience^(^ 9 ^)^; The instrument of Moral Distress which assesses the daily experience of health care personnel in various environments^(^ 12 ^)^ and the Moral Distress Questionnaire which assesses culture specific moral distress in nursing practice^(^ 13 ^)^. It is important to understand and properly evaluate moral distress originated from several effects of frequently encountered moral distress in nurses; to define the sources of stress, and to develop strategies to prevent these stress sources^(^ 14 ^)^. Despite the fact that the nurses in our country are told they will face moral distress very frequently, there are no comprehensive studies on this issue. Therefore, scales developed for this issue are needed in order to carry out scientific studies. The aim of this study was to to determine the validity and reliability of the Turkish language version of the Moral Distress Questionnaire for nurses.

## Method

The methodological objective of this study was to evaluate the validity and reliability of the Moral Distress Questionnaire (MDQ) in Nursing. It was carried out at the State Hospital in İzmir, a province located in the west of Turkey, between September 2017 and February 2018. The population of the study consists of nurses working in internal (N = 579) and surgical (N = 592) services of a university hospital.

The research sample consists of a total of 200 nurses selected by the stratified sampling method, who accepted to participate in the research and who work in the Internal (N = 579) and Surgical (N = 592) Services of a university hospital. The number of nurses required to be included from each service was calculated with the proportional stratified random sampling method by considering the total number of nurses to ensure that the internal and surgical services can be predominantly represented. Thus, a total of 200 nurses (99 from Internal Services and 101 from Surgical Services) were included in the research sample. The sample size was calculated using the Open Epi (version 2 open source) website. Taking α = 5%, effect size (*d*) = 0.30, and 1 - β (power) = 0.80 (80%), the minimum sample size was calculated as 152.

The data of the study were collected with an Individual Presentation Form and MDQ in Nursing. Individual Presentation Form: a questionnaire was developed by the researchers in accordance with the literature to determine the sociodemographic characteristics of the participants^(^ 1 ^,^ 6 ^,^ 10 ^)^. It consists of 9 questions including the age of the nurses, the unit they worked in, their working years, their responsibility in the unit, their educational status, their marital status, their status of having children, their health status and introductory information about drugs used consistently. MDQ: the Moral Distress Questionnaire is of the Likert type and contains a total of 15 items^(^ 13 ^)^. Seven of these 15 (item 3, 4, 8, 10, 11, 13, 15) of the MDQ were adopted considering the qualitative study phase; three of them (item 2, 5, 12) were adopted considering qualitative findings of the Stress of Conscience Questionnaire^(^ 9 ^)^ and the rest (item 1, 6, 7, 9, 14) were adopted considering the qualitative findings of The instrument of Moral Distress^(^ 12 ^)^. All the items of the questionnaire are positive and ranked in the range of 1-6 points as “Strongly Disagree”, “Agree”^(^ 13 ^)^.

The questionnaire consists of 3 sections: Factor 1: Relations: items 2, 5, 9, 10, 11, 12; Factor 2: Possibilities: items 3, 4, 13, 14, 15 and Factor 3: Time: items 1, 6, 7, 8. The score obtained from the questionnaire is between 15 and 90.

The data were collected by the researchers using the face-to-face interview method with the nurses who accepted to participate in the research. The data collection tools were filled in 15-20 minutes. Required permission was obtained by e-mail from Michal Mashiach Eizenber, the author of the MDQ for nursing, for which validity and credibility are to be made. Permission was obtained to conduct this study, both from the Ege University Nursing Faculty (EÜHF: 2015-42) and from the nurses participating in the research.

The data were evaluated in a computer environment [Statistical Package for the Social Sciences (SPSS) version 21.0 software for Windows and AMOS 23]. The data regarding the descriptive characteristics of the nurses were evaluated by number, percentile, mean, and standard deviation. The Content Validity Index (expert opinion) was evaluated by Kendall’s coefficient of agreement. In the scale development, exploratory factor analysis and then confirmatory factor analysis were applied. Explained variance was used for determining the factors’ structure. The Chi-square test (*χ* ^2^), degree of freedom (*df*), the ratio of *df* to *χ* ^2^ (*χ* ^2^/*df*), normed fit index (NFI), goodness-of-fit index (GFI), comparative fit index (CFI), incremental fit index (IFI), relative fit index (RFI), non-normed fit index (NNFI) and root-mean-square error of approximation (RMSEA), goodness-of-fit indexes, were assessed for this model. The correlation between the item total scores was examined using Pearson’s correlations analysis. For reliability, test-retest, Pearson’s Correlation Coefficient based on item analysis, and internal consistency analysis were performed. The reliability of the scale was tested for internal consistency and evaluated using Cronbach’s alpha reliability coefficient and item total score reliability analysis. The significance level was accepted as p < .05.

Studies regarding language validity of the MDQ scale were conducted in the first phase of the study. The English text of the MDQ Scale was given to an academic group of twelve people (ten nursing lecturers and two professors of psychiatry) with PhD titles and they were asked to translate the text into Turkish for translation study (translating-retranslating) of the scale. Afterwards, two linguists, who had never seen the English text of the scale before, translated it from Turkish into English. The items of the scale that were translated from Turkish into English were compared with the items of the original scale and the necessary corrections were made. The Turkish version of the scale was presented to a group of 12 experts and the final version of the scale was shaped with the recommendations from the experts.

The content validity study was conducted for the scale of which the language equivalence study was completed. At this stage, the experts were consulted with the purpose of determining whether the questions on the scale were appropriate for the purpose of measurement, whether they represent the field to be measured, whether they are related to the problem being addressed and whether they include different concepts outside the field^(^ 15 ^)^. The Turkish version of the scale was presented to twelve experts from different fields (nursing, psychology) with this purpose. The Content Validity Index (CVI) was used to evaluate the experts’ opinions. The level of measurement of each item was asked to be evaluated by the experts using expressions as 1 = Not suitable, 2 = The item needs to be changed as appropriate, 3 = Minor changes required, 4 = Completely appropriate. Mean, standard deviation, and median of the scores, and the lowest and the highest scores given by the evaluators were calculated. Kendall’s Agreement Coefficient (W) was calculated to measure the conformity of the scores given by the evaluators^(^ 15 ^-^ 16 ^)^. Exploratory Factor Analysis/Main Components Analysis, and Confirmatory Factor Analysis were used for factor construct validity. The suitability of the data for factor analysis was examined using the Kaiser-Meyer-Olkin (KMO) value and the Bartlett sphericity test. The high value of the KMO indicates that each variable in the measure can be perfectly predicted by other variables. It is concluded that the correlation or covariance matrix is not a unit matrix; in other words, a data set in which factor analysis can be applied is being studied if the calculated probability of error (p-value) of the Bartlett sphericity test is below 0.05^(^ 15 ^,^ 17 ^-^ 18 ^)^.

Items with a factor load value of 0.30 or higher in the Confirmatory Factor Analysis (CFA); and 0.32 or higher in the exploratory factor analysis were taken to factor constructs. Chi-Square Goodness (x2/sd), GFI, AGFI, CFI, NNFI, Standardized Root Mean Square Residuals (SRMR), and RMSEA fit indexes which have multiple fit indexes for CFA were investigated. In the fit indexes, >0.90 for GFI, AGFI and CFI and <0.05 for RMSEA and SRMR are accepted as criteria. However, a value <0.08 is taken as acceptable goodness of fit value for RMSEA and SRMR. In addition, x2/sd is required to be ≤2, and the model is considered acceptable in the situations where this value is below 5^(^ 17 ^,^ 19 ^)^. Reliability is the power of a measurement tool to present sensitive, coherent, and stable measurement results.

200 randomly selected participants were asked to find a nickname for themselves and to indicate it on the questionnaire during their first participation. The same scale was applied to the test group after 2 weeks and they were asked to re-write the same nickname. Afterwards, the questionnaires with the same name were matched and re-test results were obtained.

## Results

51.5% (n=103) of the nurses participating in the study were in the age range of 28-37 and the mean age was 31.66 ± 6.16. 50.5% (n=101) of the nurses worked in the surgical department of the hospital and had a mean working experience of 8.60 ± 6.27 years. 82.5% (n = 165) of the nurses had a Bachelor’s degree, 59.5% (n=119) were married and 41.5% (n=83) had children (Table 1).

**Table 1:** Distribution of the nurses according to demographic characteristics (n=200). İzmir, Turkey, 2017-2018

Demographic characteristics	N* (%^†^)
**Age in years (x=31.66±6.16)**	
18-27	53(26.5)
28-37	103(51.5)
38 and older	44 (22.0)
**Work department**	
Internal	99 (49.5)
Surgical	101(50.5)
**Working experience in years (X=8.60±6.27)**	
1 - 4	62 (31.0)
5-9	61 (30.5)
10-14	37 (18.5)
15 and more	40 (20.0)
**Educational status**	
Master's degree	6 (3.0)
Bachelor's degree	165(82.5)
Associate degree	16 (8.0)
Vocational school of health	13 (6.5)
**Marital status**	
Married	119(59.5)
Single	75 (37.5)
Widowed/Divorced	6 (3.0)
**Status of having children**	
Yes	83 (41.5)
No	117(58.5)
**Status of having a health problem**	
Yes	43 (21.5)
No	157(78.5)

* N = Number;

† % = Percentage

Kendall’s Agreement Coefficient correlation test was conducted to examine the content validity of the scale. It was determined that there was a significant level of fit among the experts when the content validity of 15 items on the scale and the evaluating scores of the experts were evaluated by W analysis (W=0.50, p <0.05). In order to assess the reliability of this 15-item scale, the item–total correlations, Cronbach’s alpha, and the test–retest method were used. A positive and highly significant correlation was found between the first measurement and the second measurement as a result of the test–retest method which was conducted to demonstrate that the scale developed made coherent measurements (r=.98, p<.05).

**Figure 1:**
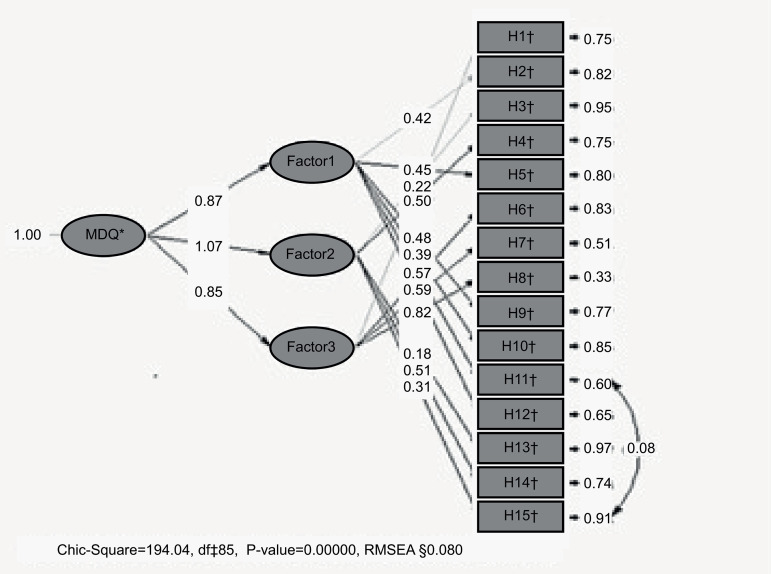
Moral Distress Questionnaire confirmatory factor results. İzmir, Turkey, 2017-2018 ^*^MDQ = Moral Distress Questionnaire; ^†^H = Questionnaire; ^‡^df = Degree of freedom; ^§^RMSEA **=** Root mean square error of approximation

The KMO value was calculated before factor analysis was performed, and was found to be 0.77. Additionally, the Bartlett sphericity test results were calculated as X² (200) = 645.245 (p<0.001). These results show that a data set which was appropriate for factor analysis was used. As a result of this analysis, the eigenvalue of three factors was found to be above 1 and it was seen that these three factors explained 44.92% of the total variance. According to the confirmatory factor analysis, the factor loads for the model are shown in Figure 1.

The significance p value gives information about the difference (value) between the observed covariance matrix and the expected covariance matrix. The p value is expected to be significant in CFA^(^ 17 ^,^ 19 ^)^. The values on the right side of the figure show the error variances of each item and the values in the middle of the figure show the factor loads. It is seen that error variances have values of 0.97 and below when the error variances of the variables are examined. It was found that the Chi-square value (X^2^=194, n=200, sd=85, p=0.001) was significant. The fit indexes of the model are given in Table 2.

**Table 2:** Findings regarding confirmatory factor analysis. İzmir, Turkey, 2017

Index	Perfect Fit Criterion	Acceptable Fit Criterion	Finding of Research	Result
	0-3	3-5	2.28	Perfect fit
RMSEA^†^	.00 ≤ RMSEA^†^ ≤ .05	.05 ≤RMSEA^†^ ≤ .10	.08	Good fit
CFI^‡^	.95 ≤CFI^‡^ ≤ 1.00	.90 ≤CFI^‡^ ≤ .95	.88	Good fit
NNFI^§^	.95 ≤NNFI^§^ ≤ 1.00	.90 ≤NNFI^§^≤ .95	.86	Good fit
SRMR^‖^	.00 ≤ SRMR^‖^ ≤ .05	.05 ≤SRMR^‖^ ≤ .08	.07	Good fit
GFI^¶^	.95 ≤GFI^¶^ ≤ 1.00	.90 ≤GFI^¶^ ≤ .95	.88	Good fit
AGFI**	.90 ≤ AGFI** ≤ 1.00	.85 ≤AGFI** ≤ .90	.85	Good fit

Source: Yilmaz, 2018^(^17^)^

*χ^2/sd = Chi-square goodness;

† RMSEA = Root mean square error of approximation;

‡ CFI = Comparative fit index;

§ NNFI = Non-normed fit index;

‖ SRMR = Standardized root mean square residuals;

¶ GFI = Goodness of fit index;

** AGFI = Adjusted goodness of fit index

The fit index, which should be examined first in CFA, is Chi-square (X^2^) fit statistic, and it says that if the ratio to the degree of freedom is less than 3, it shows perfect fit; and if it is below 5, it shows good fit^(^ 20 ^)^. This ratio was found to be 2.28. RMSEA is the square root of the mean of error squares and it says that, in order for the model to be significant, a value below 0.05 means perfect fit and a value below 0.10 means good fit^(^ 20 ^-^ 22 ^)^. The RMSEA value was found to be 0.08 and it shows good fit (Table 2). CFI is a fit index that compares the covariance matrix predicted by the model with the covariance matrix of the null hypothesis model^(^ 17 ^-^ 20 ^)^. The CFI takes values ranging between 0 and 1. It can be concluded that a model with a CFI value between 0.95 and 1 has a good fit and a model with a CFI value between 0.90 and 0.95 has an acceptable fit^(^ 17 ^,^ 20 ^-^ 22 ^)^. Some researchers have taken the value of 0.80 as a more flexible limit^(^ 23 ^)^. Although the CFI (0.88) and NNFI (0.86) values calculated for the best model that can be established in this study are below the generally accepted value, it can be said that the model is acceptable due to its complexity (Table 2).

The GFI shows the amount of general covariance between the variables calculated and observed by the assumed model. The GFI value ranges from 0 to 1. It is considered as a good model if the GFI value exceeds 0.90. This means that enough covariance has been calculated among the observed variables^(^ 20 ^)^. The GFI value was found to be 0.88, and it indicates good fit. In addition, AGFI means adjusted fit index and it was found to be 0.85, indicating good fit (Table 2). SRMR means standardized root mean square residuals. The closer the SRMR value is to 0, the better the fit of the model. If the model has a SRMR value lower than 0.05, it indicates good fit; and if it has a SRMR value between 0.05 and 0.08, it indicates acceptable fit^(^ 20 ^-^ 22 ^)^. The value of 0.07 found in the study indicates acceptable fit. The scale was found to be acceptable with the goodness of fit values obtained in the Confirmatory Factor Analysis. Cronbach’s alpha coefficient was found to be 0.79 as a result of the analysis conducted in order to test the internal consistency of the scale. On the other hand, the Cronbach’s alpha values of the sub-dimensions of the scale were calculated as .64 for Factor 1; .44 for Factor 2 and .69 for Factor 3.

## Discussion

This methodological research was conducted with the aim of investigating the validity and reliability analyses of the MDQ which was developed to determine the moral distress experienced by nurses, for the Turkish society. In this research, it was found that the experts reached an agreement in terms of the content of the items as a result of the Kendall’s Agreement Coefficient Correlation Test which was conducted for the content validity of the MDQ in Nursing.

Pearson’s Correlation Coefficient between the first and the second implementation of the MDQ in Nursing for test-retest validity was determined to be 0.98, and a statistically significant correlation was found (r=.98, p <.05). The test-retest approach is used to predict the invariance of a tool over time; while higher coefficients are obtained in short-interval measurements, lower reliability coefficients may occur in long-interval measurements due to variations^(^ 16 ^,^ 24 ^)^. The very high level of correlation coefficient between the first and second implementation demonstrates the reliability of the participants’ responses.

The exploratory factor analysis method, which was one of the factor analysis methods suggested in the literature^(^ 25 ^-^ 26 ^)^, was used to evaluate the construct validity for the scale development study. In the literature^(^ 25 ^-^ 26 ^)^ it is stated that, as a result of the sampling adequacy test result, the KMO value should be, at least, 0.50; a value between 0.50-0.60 is considered bad; a value between 0.60-0.70 is considered weak; a value between 0.70-0.80 is considered fair; a value between 0.80-0.90 is considered good and a value higher than 0.90 is considered perfect, when the factor analysis method is used^(^ 15 ^,^ 18 ^,^ 22 ^)^. The KMO value was found to be 0.77 for the MDQ. It was concluded that the sampling size was moderately enough for factor analysis. The decision on whether the structure would be divided into factors or not after the adequateness of the sampling size is analyzed with the Bartlett sphericity test. It is stated that the factors can only be revealed when the significance value obtained from the analysis is less than 0.05^(^ 16 ^,^ 22 ^,^ 26 ^)^. In the Bartlett sphericity test analysis of the study, p<0.001 was found. For this reason, it was determined that the structure of the MDQ could be divided into factors. The fact that the confirmatory factor analysis results are statistically significant indicates that the scale can be accepted according to the calculated goodness of fit values and that the structure of the MDQ, with three factors composed of 15 items, is confirmed as a model.

It is stated in the literature^(^ 19 ^,^ 22 ^,^ 25 ^-^ 26 ^)^ that the Cronbach’s alpha coefficient changes between 0 and 1; a coefficient value between .60 and .80 indicates that the scale is very reliable and a coefficient value of .80 and above indicates the scale is perfectly reliable. In this study, the Cronbach’s Alpha coefficient of the Turkish form of the scale is 0.79, and it indicates that the reliability of the scale is high for measuring Moral Distress in nurses. The Cronbach’s alpha values of the sub-dimensions of the scale were calculated as .64 for Factor 1, .44 for Factor 2 and .69 for Factor 3. It was seen that the Cronbach’s alpha values did not generally increase when any of the items were subtracted as a result of the item analysis of the MDQ. This situation was evaluated positively in terms of the reliability of the items and no addition or subtraction process was carried out on the items of scale^(^ 18 ^)^. The Cronbach’s alpha values of the sub-dimensions of the MDQ found were .85 for Factor 1, .79 for Factor 2, and .80 for Factor 3^(^ 13 ^)^.

This methodological study was carried out in only one university hospital in İzmir, in a region of Western Turkey. The results cannot be generalized.

## Conclusion

As a result of the confirmatory factor analysis conducted to test the construct validity, it has been found that the MDQ is composed of three factorial constructs and that the model data fit for the construct validity is acceptable. The findings of the MDQ as a result of internal consistency calculations for total and sub-dimensions show that the reliability of the scale is high. The analyses made have shown that, despite the low reliability results of some of the items, the MDQ was found to be a valid and reliable tool to determine the moral distress experienced by nurses in the sampling of Turkish professionals.

This scale is an evaluation tool that can be quickly and easily used to determine the moral difficulties experienced by nurses. For this reason, it is suggested that the MDQ should be applied to a wider sampling group of individuals from different health care departments.
